# An Update on Tissue Contraction in Immediate Breast Reconstruction Using an Adjustable Implant Partially Filled With Air

**DOI:** 10.7759/cureus.33463

**Published:** 2023-01-06

**Authors:** Payton Yerke Hansen, Oscar A Vazquez, Savannah Braud, Jacob Komberg, Samuel A Mansour, Hilton Becker

**Affiliations:** 1 Department of Surgery, Florida Atlantic University Charles E. Schmidt College of Medicine, Boca Raton, USA; 2 Department of Surgery, Stanford University, Palo Alto, USA; 3 Internal Medicine, Los Robles Health System, Thousand Oaks, USA; 4 Department of Surgery, Detroit Medical Center/Wayne State University, Detroit, USA

**Keywords:** plastic and reconstructive surgery, reconstructive surgery, aesthetic surgery, breast surgery, plastic surgery

## Abstract

Background

With the use of nipple-sparing mastectomy (NSM) and skin-sparing mastectomy (SSM) techniques, utilizing a prepectoral underfilled adjustable saline implant allows for tissue contraction and thickening of the flap. This procedure allows for immediate reconstruction using an adjustable implant as a spacer with less risk of skin flap compromise and improves cosmetic outcomes.

Methods

A retrospective chart review of patients presenting to a single surgeon from September 2013 to September 2021 for breast reconstruction utilizing an underfilled prepectoral adjustable implant following SSM or NSM was performed. Baseline patient demographics, clinical information, postoperative complications, conversion to silicone implant, and contraction distance were recorded and analyzed.

Results

Fifty-four patients underwent prepectoral breast reconstruction using a Spectrum™ adjustable implant following an NSM or SSM. Tissue contraction and skin flap elevation were observed in all patients. The amount of tissue contraction averaged 4.4 cm (mean). Seven patients (12.96%) developed seromas. Four patients (7.41%) developed hematomas in the early postoperative period. Two patients (3.67%) developed capsular contracture. Two patients (3.67%) had a displaced port. After converting the air to saline, 25 patients (46.29%) opted for a secondary reconstructive procedure to exchange the saline implants for silicone gel implants.

Conclusion

Tissue contraction reduces the need for skin excision in ptotic breasts undergoing reconstruction procedures following NSM or SSM. The partially filled implant initially functions as a spacer to prevent flap adherence to the pectoral muscle and minimizes tension on the flap to promote flap thickening, elevation around the underfilled implant, and maximizes breast projection and overall aesthetic outcome.

## Introduction

Breast cancer is the most frequently diagnosed malignancy in women, and the rates are projected to increase. However, advancements in treatment have vastly improved clinical outcomes in breast cancer patients. With more women being diagnosed with breast cancer and a higher survival rate, mastectomies followed by breast reconstruction are commonly used [1]. Reconstruction can occur either immediately after the mastectomy or at a later date through the use of autologous tissue flaps or prosthetic implants [2,3]. These procedures commonly involve tissue expanders to stretch the skin in preparation for the implant [4].

Delayed reconstruction usually results in decreased surgical complications [2]. During the healing process after a mastectomy, delayed reconstruction also allows for the skin flap to naturally contract and thicken, especially if it is thin after the procedure and/or radiation. This causes the tissue to elevate and better adhere to the pectoral muscles underneath, which results in an increased breast projection [5]. Despite this, immediate reconstruction has become more conventional because of increased aesthetic outcomes and patient satisfaction [6].

With the transition to immediate reconstruction, skin-sparing mastectomy (SSM) and nipple-sparing mastectomy (NSM) were introduced to further enhance the cosmetic outcome. In SSM, most of the breast skin is preserved. This creates a pocket that can be filled with an autologous graft or implant during reconstruction. In NSM, the skin and the nipple-areola complex (NAC) are conserved [4]. With the preservation of excess skin, tissue expanders are no longer necessary.

While it is standard procedure to use tissue expansion for simple mastectomies in the prepectoral plane, the utilization of an underfilled Spectrum™ (Mentor Worldwide LLC, Santa Barbara, USA) implant allows for postoperative tissue contraction and greater control over the healing aesthetics. With the use of NSM and SSM techniques, we have found that mastectomy flaps will contract, similar to what is observed in delayed reconstruction when an underfilled implant is used. When the implant is filled with air instead of saline, there is tension pressure placed on the inferior flap due to the lighter weight. This allows for tissue contraction and thickening of the flap, which elevates the breast. This procedure also allows for immediate reconstruction using an adjustable implant as a spacer with minimal risk of skin flap compromise and capitalizes on the natural ability of the skin to contract following breast reconstruction [5]. We present a retrospective analysis of 54 patients in which skin contraction was utilized to improve breast reconstruction outcomes with longer follow-up and contraction measurements. This study provides important insight into a new surgical technique that can be utilized to enhance the post-reconstructive aesthetics of ptotic breasts.

## Materials and methods

Written informed consent was gathered from the patients, and the ethical principles outlined in the 2013 Declaration of Helsinki were strictly followed. A retrospective analysis of patients who underwent an SSM or NSM followed by breast reconstruction using a pre-pectoral adjustable Spectrum™ (Mentor Worldwide LLC, Santa Barbara, USA) implant was performed. All patients were seen by a single plastic surgeon ranging from 2014 to 2021. The mean reconstruction time was 24.3 months (+/- 4 months). Details regarding patients' past medical history, operative technique, and postoperative outcomes were gathered from provider clinical notes.

A total of 54 cases of breast reconstruction using a prepectoral adjustable Spectrum™ implant following an SSM or NSM are described. Patients who had ptotic breasts were considered optimal candidates for this procedure. Additionally, patients who underwent sentinel lymph node biopsy or axillary lymph node dissection were included in this study. We analyzed data including patient age, preoperative diagnosis, initial implant volume, final implant volume, before and after nipple to sternal notch measurements, length of the reconstruction process, additional procedures, and complications.

Surgical technique

The preferred candidates for this procedure were women with large, ptotic breasts undergoing immediate breast reconstruction following SSM or NSM. Patients in this study ranged from ptosis 2-3. There was no breast shape criteria. The procedure is contraindicated in two-stage reconstruction patients.

After the mastectomy was performed by a breast surgeon, the lateral and axillary pockets were closed using a running No. 1 STRATAFIX™ (Ethicon, Bridgewater, USA) suture. A smooth Spectrum™ adjustable saline implant was placed in the prepectoral space. In some cases, long-term absorbable monofilament DuraSorb™ (Surgical Innovation Associates, Chicago, USA) or GalaFLEX® (Galatea Surgical, Lexington, USA​) mesh was used to support the upper pole. Air was injected through the removable injection port using a syringe filter (Cole-Parmer, Vernon Hills, USA) to prevent contamination of the implant. It was initially underfilled with air. The amount of air used varied based on implant size to prevent implant collapse. The amount of air ranged from 0% to 88% (mean of 35.8%) of the total implant volume. The breast was taped in an elevated position with a Tegaderm™ (3M, Saint Paul, USA) dressing. Postoperatively the Tegaderm™ (3M, Saint Paul, USA​​​​) was reinforced or replaced with paper tape. 

The tissue was allowed to heal and naturally contract over the underfilled implant. Close postoperative follow-up was performed in all cases. Additional contraction could be obtained by filling the implant with the lowest volume of air permitted to prevent implant collapse. This was done using a 23-g butterfly needle inserted into the injection port. The air could be replaced with saline once the desired cosmetic result is reached by aspirating the air through the injection port with a 23-g butterfly needle and 60-cc syringe. The first saline injection occurred on average 16.1 days (mean) postoperatively. The final saline injection averaged 299.6 days (mean) postoperatively. The injection port was then removed under local anesthesia. Implant rippling was occasionally seen after the injection of saline, but it can be corrected with fat injections or replacement with a silicone gel implant at the patient's request. An acellular dermal matrix (ADM) was not used on patients receiving fat injections. Instead, platelet-rich fibrin was used because it provides a structural matrix to support adipocytes. A second procedure is required for patients who want to replace the implant with silicone gel implants. 

## Results

Fifty-four patients (94 breasts) underwent a prepectoral breast reconstruction using a Spectrum™ adjustable implant. Thirty-two (59.3%) patients underwent NSM, and 22 (40.7%) had an SSM. Additionally, 10 (18.52%) were diagnosed with ductal carcinoma in situ (DCIS), 28 (51.58%) were diagnosed with invasive ductal carcinoma (IDC), 12 (22.22%) were diagnosed with invasive lobular carcinoma (ILC), and four (7.47%) underwent a prophylactic mastectomy. The mean age was 56.1+/-12.4, and the average BMI was 26.1+/-5. Forty patients (74.07%) had a bilateral mastectomy followed by immediate reconstruction, and 14 patients (25.93%) underwent a unilateral mastectomy and contralateral breast reduction. The implant size ranged from 225 cc to 575 cc (mean of 393 cc). Initially, the implants were underfilled with air. The amount of air ranged from 0% to 88% (mean of 35.8%). Tissue contraction and skin flap elevation were observed in all patients. We were able to measure the amount of elevation in 20 patients. All measurements were taken from the sternal notch to each nipple, with the patient sitting upright. The preoperative measurement ranged from 18 cm to 35 cm (mean of 25.7 cm). One year postoperatively, the measurements ranged from 16 cm to 27 cm (mean of 21.1 cm). Overall, the amount of tissue contraction ranged from 0 cm to 14 cm (mean of 4.4 cm). This is further demonstrated in Table 1. 

**Table 1:** Patient characteristics

Characteristic	Overall
Number of patients	54
Mean age (years)	56.06
Nipple-sparing mastectomy (%)	59.30
Skins-sparing mastectomy (%)	40.70
Current smoker (%)	5.60
Former smoker (%)	14.80
Chemotherapy (%)	9.30
Mean BMI (kg/m^2^)	26.1
Mean implant size (cc)	393.3
Mean implant fill, left (%)	36.65
Mean implant fill, right (%)	34.94
Average implant fill (%)	35.79
No. of breasts	94
Seromas	7 (12.96%)
Hematomas	4 (7.41%)
Capsular contracture	2 (3.70%)
Displaced injection port	2 (3.70%)
Partial necrotic nipple	9 (16.67%)
Implant exchange to silicone	25 (46.29%)
Fat grafting after implant removal	4 (7.41%)
Complete removal	2 (3.70%)
Mean left contraction (cm)	4.45
Mean right contraction (cm)	4.26
Total average contraction (cm)	4.36
Minimum contraction (cm)	0
Maximum contraction (cm)	14
Average reconstruction (days)	274.14
Average reconstruction (months)	4.68
Breast cancer diagnosis	52 (96.29%)
Bilateral mastectomy	40 (74.07%)
Unilateral mastectomy	14 (25.93%)
Mean follow-up (months)	24.33

A small amount of air can diffuse through the expander, but weekly follow-ups did not show any complications related to air diffusion. Seven patients developed seromas (12.96%). All seromas were successfully aspirated using a Blunt SeromaCath™ (Greer Medical, Santa Barbara, USA) [7]. Four patients (7.41%) developed hematomas in the early postoperative period. All hematomas were successfully evacuated. Two patients (3.70%) developed capsular contracture, both Baker 2 in classification. Two patients (3.70%) had a displaced port by visual inspection. Nine patients (16.67%) had partial nipple necrosis or wound edge necrosis. They were all successfully treated by reducing the volume of the implant and debriding the necrotic tissue, and performing secondary closure. Volume reduction or even temporarily emptying the implant allowed for tension-free closure. Once the healing was assured, serial filling of the implant was instituted. One patient (1.85%) opted for a bilateral prophylactic mastectomy due to a positive BReast CAncer gene 2 (BRCA2) genotype. Additionally, one patient (1.85%) had atypical ductal proliferation.

After converting the air to saline, 29 patients kept their Spectrum™ implants. Additionally, 25 patients (46.29%) opted to undergo a secondary reconstructive procedure to exchange the saline implants for silicone gel implants. The mean size of the silicone implants was 415.7 cc. Four patients (7.41%) had their Spectrum™ implants removed, and breast volume was attained through fat grafting. Additionally, two patients (3.70%) had their saline implants removed with no additional procedures. These patients were able to undergo the secondary procedure a minimum of three months after their initial reconstruction to allow for proper wound healing. Further, patients were stratified into radiation versus no radiation groups, as indicated in Table 2. Twenty-four (44.44%) patients had radiation treatment. There was no significant (p>0.05) difference in the tissue contraction measured between the patients who underwent radiation and those who did not. All patients were pleased with their results.

**Table 2:** Radiation vs. no radiation sub-groups

Characteristic	Radiation (%)	No radiation (%)
No. of patients	24 (44.44)	30 (55.56)
Mean age (years)	54.33	57.37
Mean BMI (kg/m^2^)	26.7	25.6
No. of breasts	42 (44.68)	52 (55.32)
Seromas	4 (16.67)	3 (10)
Hematomas	1 (4.17)	3 (10)
Capsular contracture	2 (8.33)	0
Displaced injection port	0	2 (6.67)
Partial necrotic nipple	4 (16.67)	5 (16.67)
Implant exchange to silicone	9 (37.5)	16 (53.33)
Fat grafting after implant removal	2 (8.33)	2 (6.67)
Complete removal	1 (4.17)	1 (3.33)
Mean left contraction (cm)	4.33	4.55
Mean right contraction (cm)	3.78	4.7
Mean contraction (cm)	4.06	4.62
Bilateral mastectomy	18 (75)	22 (73.33)
Unilateral mastectomy	6 (25)	8 (26.67)
Minimum contraction (cm)	0	0
Maximum contraction (cm)	9	14
Mean follow-up (months)	23.88	24.7

## Discussion

Tissue contraction can be utilized in breast reconstruction because it relies on the natural ability of myofibroblasts to contract the tissue following tissue injury. Previous procedures following radical mastectomies necessitated the use of a subpectoral tissue expander to stretch the overlying muscle and skin to create a breast mound. However, with the introduction of skin-sparing mastectomies and prepectoral reconstruction, tissue expansion is rarely needed [8]. Rather, these procedures often result in excess skin that is surgically excised. Tissue contraction produces a mastopexy effect, so a two-stage procedure can be avoided. Additionally, skin contraction promotes the elevation and thickening of the skin flap, which allows for better implant coverage and improved aesthetic outcomes without the need for an acellular dermal matrix (ADM). Utilizing an adjustable implant without ADM significantly reduces the postoperative complication rate. While ADM has been shown to reduce capsular contracture, seroma and infections are more serious complications that should be considered [5,9].

With tissue contraction, the subcutaneous tissue of the mastectomy flap initially contracts due to myofibroblast involvement. Tissue contraction occurs both as a result of the decrease in weight and the action of elastin fibers. Skin contraction is commonly observed in skin-sparing mastectomies without implant placement. As the loose skin contracts, it adheres to the underlying muscle. Additional contraction occurs throughout the healing process until the myofibroblasts associate with collagen extracellular matrix and revert back to fibroblast phenotype. This same transformation is seen when there is contact between the exposed flap and the surface of an implant. When the implant is smaller than the pocket, contraction occurs until the excess space is obliterated. 

In our study, prepectoral breast reconstruction is achieved using a Spectrum™-adjustable saline implant. The implant is placed at the time of the mastectomy and functions as a spacer to prevent the flap from adhering to the muscle. Initially, the implant is underfilled with air, and it disperses evenly throughout the entire lumen rather than pooling at the lower pole, which is commonly seen in underfilled saline-filled implants. This prevents flap adhesion to the chest wall and thus reduces the need for delayed reconstruction [10,11]. Since the implant is only filled with air, it is lightweight and exerts low amounts of tension. This phenomenon promotes the tissue's natural ability for contraction without excessive pressure on the lower flap, which is commonly seen in saline-filled expanders and gel implants. By decreasing the lower flap pressure, vascular compromise can also be avoided. In the event of vascular compromise leading to skin flap necrosis or wound dehiscence, the implant can be completely emptied, which reduces all the tension and pressure on the flaps. This facilitates debridement and successful secondary closure. 

**Figure 1:**
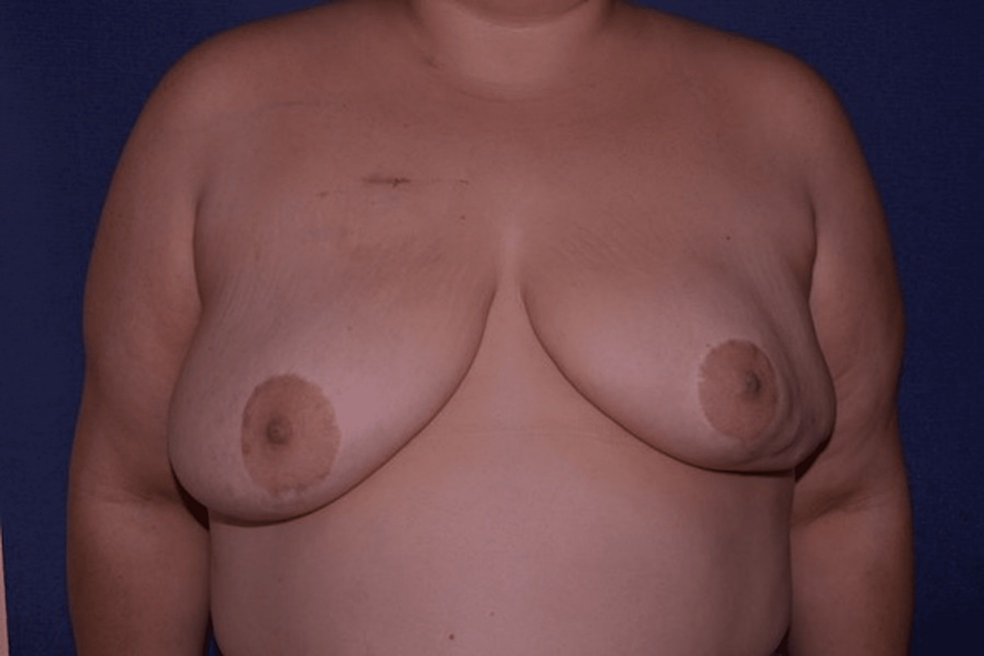
Preoperative image of a 42-year-old female

**Figure 2:**
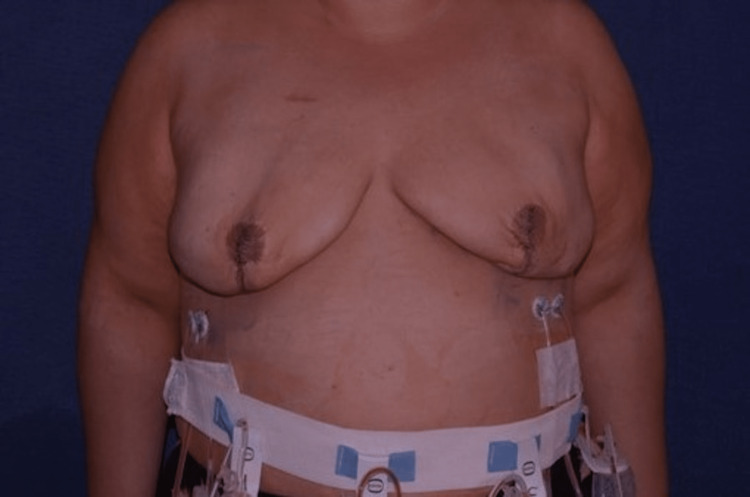
Image of a 42-year-old female on postoperative day seven The breast implants are underfilled with air.

**Figure 3:**
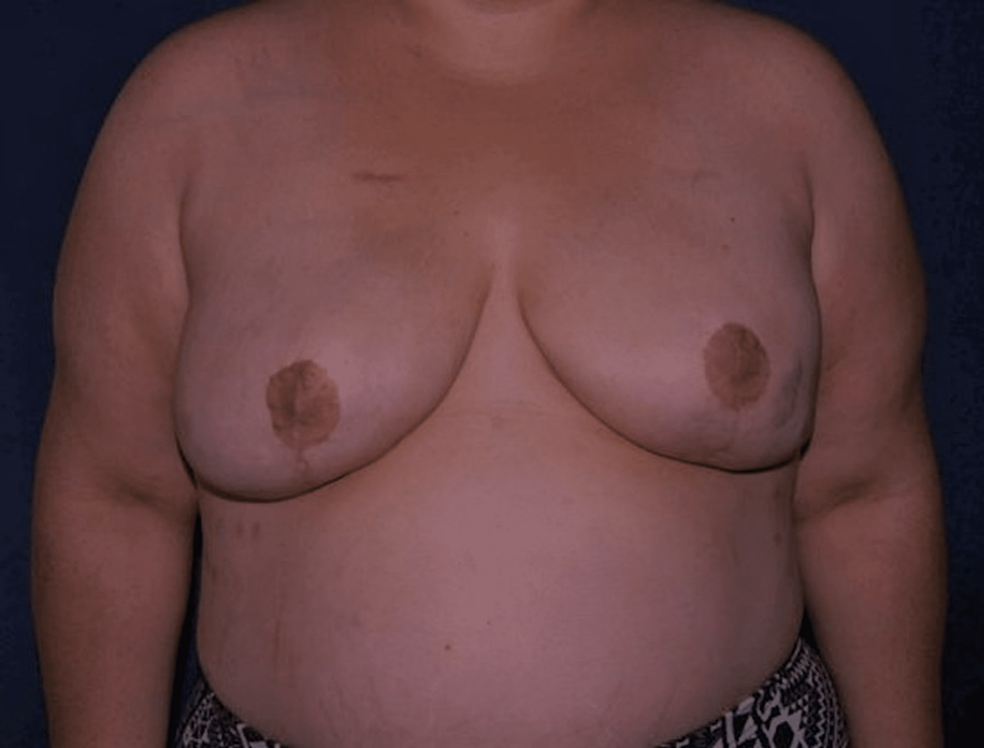
Image of a 42-year-old female at two years postoperatively The implants are now filled with saline.

An important advantage of underfilling the adjustable implant with air is it can be used to control the amount of contraction by adjusting the volume within the implant. Underfilling the implant will result in additional contraction. This occurs until the exposed surface is in contact with the implant. Excessive contraction can be prevented by filling the implant with more saline to add additional weight. Furthermore, the position of the implant can be modified at various stages postoperatively. If the implant is sitting too low, the surgeon can reduce the volume of air in the implant and place a strap inferiorly for approximately one to two weeks (Figures 1-3). This will elevate the implant, which can then be filled with saline to achieve the desired aesthetics. On the other hand, if the implant is too high, the air can be replaced with saline, and a strap can be placed superiorly to lower the implant. In comparison, expanders with fixation patches do not have the ability to be repositioned postoperatively. The versatility of this technique allows for improved control to achieve the desired cosmetic outcome.

The underfilled adjustable implant allows the skin flap to thicken naturally, which reduces the need for ADM. This results in a lower rate of skin rippling. However, some patients may require fat injections to obtain their desired cosmetic result. The underfilled implant is also lighter and more comfortable for patients, which increases the postoperative acceptance rates. A downside to this technique is premature skin contraction around the implant. To prevent this, frequent clinical follow-ups are required to control the degree of contraction around the implant. 

Additionally, this surgical technique was utilized on a variety of breast cancer subtypes in our patient population. While most of our patients had IDC, DCIS poses a difficulty to surgeons. DCIS oftentimes does not progress to invasive carcinoma. However, it has high rates of life-threatening recurrences following breast-conserving surgeries [12]. Further research can investigate this technique on micropapillary carcinomas. Micropapillary carcinomas are a rare histological subtype of breast cancers that comprises 2-8% of all diagnosed breast cancers. It is noted to have a higher histological grade and rate of lymph node metastasis. Furthermore, micropapillary carcinomas have larger tumors, which presents an oncological and surgical challenge [13,14].

While this study provides important insights, it is not without limitations. The study is a retrospective analysis with a relatively small sample size. Furthermore, the mean follow-up was 24.3 months (range: 2-37 months). Long-term evaluation to further assess the durability and postoperative results is needed. Finally, no objective cosmetic assessments or comparisons to other surgical procedures were performed. However, these parameters are not within the scope of this study. This study was designed to expand with longer follow-up and measurements on a surgical technique previously introduced that utilizes an underfilled saline implant to promote natural tissue contraction in immediate breast reconstruction cases following mastectomy. 

## Conclusions

Tissue contraction is a beneficial option for immediate breast reconstruction that warrants further investigation. By utilizing tissue contraction, the need for skin excision or two-stage procedures following skin-sparing and nipple-sparing mastectomies is reduced. The partially filled implant initially functions as a spacer and prevents flap adherence to the pectoral muscle. It also minimizes tension on the skin flap, reducing ischemic complications. Underfilling of the implant promotes flap thickening without the use of acellular dermal matrices. Furthermore, tissue contraction allows for controlled flap contraction and elevation around the underfilled implant to maximize the breast projection and overall aesthetic outcome.
